# Embolic ST-Elevation Myocardial Infarction in Atrial Fibrillation: Report of Two Cases and Review of Literature

**DOI:** 10.7759/cureus.24705

**Published:** 2022-05-03

**Authors:** Andrew V Doodnauth, Pratik Mondal, Safi Afzal, Ayesha Abdul, Vaibhavi Uppin, Samy I. McFarlane

**Affiliations:** 1 Internal Medicine, SUNY Health Science Center, Brooklyn, USA; 2 Interventional Cardiology, SUNY Health Science Center, Brooklyn, USA; 3 Internal Medicine, Campbell University - Cape Fear Medical Center, Fayetteville, USA; 4 Internal Medicine, SUNY Health Science Center, Brooklyn , USA; 5 Cardiology, SUNY Health Science Center, Brooklyn, USA; 6 Internal Medicine and Endocrinology, SUNY Health Science Center, Brooklyn, USA

**Keywords:** thromboembolic event, atrial fibrillation, coronary embolism, : acute coronary syndrome, acute st-elevation myocardial infarction

## Abstract

Coronary artery plaque rupture, erosion, thrombosis, and dissection account for nearly all acute myocardial infarction (AMI). However, coronary artery embolism remains a significant cause of AMI that is essentially unaccounted for. In this report, we present two cases of acute coronary syndrome caused by coronary embolism. Both cases illustrate that patients with atrial fibrillation are at an increased risk of thromboembolic events of the coronary circulation. We highlight the clinical characteristics of atrial fibrillation associated with coronary embolism and present the therapeutic interventions based on our experience and a review of the literature. Given that AMI is a significant cause of morbidity and mortality among adults worldwide, it is imperative that practicing clinicians be aware of coronary embolism as a cause of AMI, particularly in high-risk populations such as those with atrial fibrillation.

## Introduction

Acute myocardial infarction (AMI) secondary to a coronary artery embolism was first reported in 1856 by the famous German physician Rudolf Virchow. Coronary artery embolism accounts for approximately 3% of acute coronary syndromes (ACS), with atrial fibrillation thought to be the most common etiology [1]. In this report, we discuss two cases of ACS caused by coronary emboli. Case one describes an 80-year-old male who presented with ST-segment elevation myocardial infarction (STEMI) in the setting of paroxysmal atrial fibrillation. Case two describes a 91-year old female who presented with a non-ST-segment elevation myocardial infarction (NSTEMI). In both cases, coronary angiogram showed a thrombus occluding the culprit vessel in an otherwise non-obstructive coronary disease anatomy from atherosclerotic disease. The primary objective of this report is to highlight an underdiagnosed cause of AMI, identify general characteristics of coronary artery embolism, its association with atrial fibrillation, and discuss the most appropriate treatment strategy.

## Case presentation

Case 1

An 80-year-old African American male was brought to the nearest emergency department (ED) by emergency medical services (EMS) for acute onset of chest pain and shortness of breath. Past medical history was significant for cerebrovascular accident (CVA) with no residual deficits, insulin-dependent diabetes mellitus, hypertension, and dementia. The patient’s chest pain awoke him from his sleep five hours before arrival at the ED and felt like pressure, non-radiating, and 10/10 on a pain scale.

Initial vitals were as follows: blood pressure 192/110 mmHg, heart rate 71 beats/minute, respiratory rate 18 cycles/minute, SpO_2_ 94% on 3L nasal cannula, temperature 98.4 °F. On the physical exam, the patient was alert, oriented, and noted to be in mild distress. The patient had a regular heart rhythm, and no murmurs were appreciated. Inspiratory crackles were heard at the bases bilaterally on auscultation.

**Figure 1:**
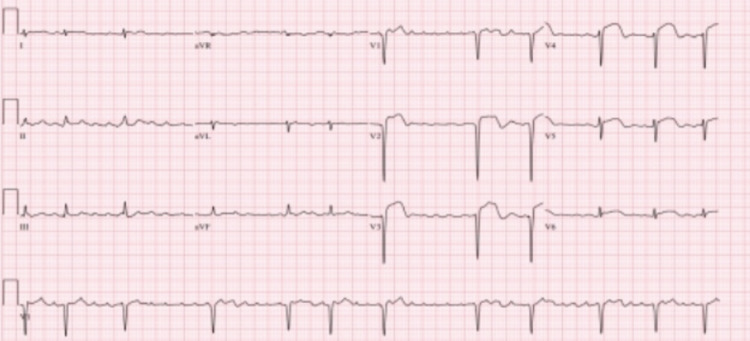
EKG, atrial fibrillation with deep Q-wave and ST-elevations in precordial leads V2-V4.

**Figure 2:**
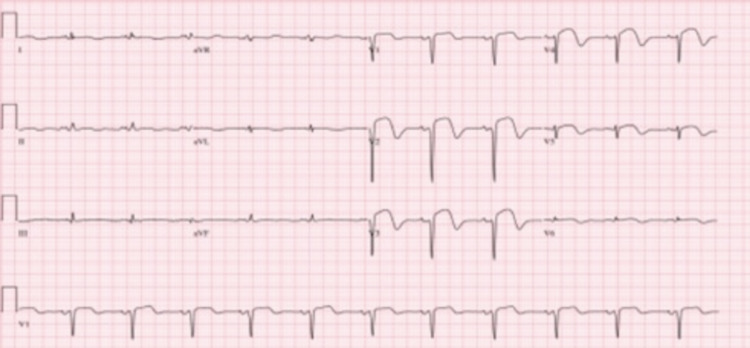
EKG, normal sinus rhythm with deep Q-wave and ST-elevations in precordial leads V2-V4.

Initial electrocardiogram (EKG) performed by EMS in the field showed atrial fibrillation with deep Q-wave and ST-elevations in precordial leads V2-V4 (Figure 1). Repeat EKG in the ED showed normal sinus rhythm (NSR) (Figure 2). The patient was taken to the cardiac catheterization lab for percutaneous coronary intervention (PCI), given his clinical presentation and EKG changes consistent with a (STEMI). Initial labs (Table 1) demonstrated an elevated lactic acid and brain natriuretic peptide, along with rising troponins. 

**Table 1:** Laboratory Values; Case Presentation 1

Case #1			
Hospital Day #	Day 1	Day 2	
Lab Value	-	-	Reference Range
White Blood Cell (WBC)	7.17	6.81	3.50 - 10.80 K/uL
Hemoglobin (Hg)	11.5	10.1	14 - 18 g/dL
Platelet Count (Plt)	238	205	130 - 400 K/uL
Serum Sodium (Na^+^)	137	140	136 - 145 mmol/L
Serum Potassium (K^+^)	5.1	4.7	3.5 - 5.1 mmol/L
Serum Chloride (Cl^-^)	102	108	98 - 107 mmol/L
Serum Magnesium (Mg^+2^)	2.2	-	1.9 - 2.7 mg/dL
Serum Calcium (Ca^+2^)	9.2	8.8	8.2 - 10 mg/dL
Serum Bicarbonate (HCO3^-^)	22	23	21 - 31 mmol/L
Blood Urea Nitrogen (BUN)	53	44	7 - 25 mg/dL
Serum Creatinine (Cr)	2.4	1.8	0.7 - 1.3 mg/dL
Troponin-I (TNI)	21.35	44.35	< 0.15 ng/dL
Brain Natriuretic Peptide (BNP)	291	-	< 100 pg/mL
Serum Lactic Acid	3.5	1.6	0.5 - 2.2 mmol/L
Venous Blood Gas pH (VBG)	7.36	-	7.31 - 7.41
Serum D-Dimer	251	-	< 499 ng/mL
Thyroid-Stimulating Hormone (TSH)	1.66	-	0.38 - 4.70 uIU/mL

**Figure 3:**
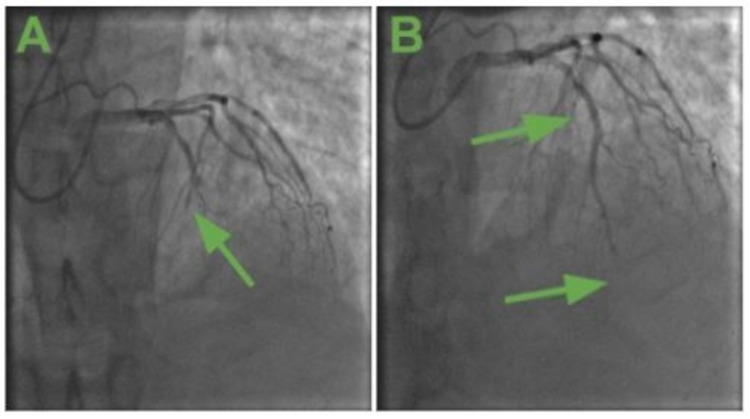
3A: (left), coronary angiography revealed 100% occlusion of the mid-left anterior descending artery (mLAD) (green arrow); 3B: (right), distal embolization of thrombus in the distal and apical left anterior descending artery and persistent no-flow phenomenon (lower green arrow) despite aggressive balloon angioplasty (upper green arrow) and intracoronary vasodilators.

In the ED, the patient received 325mg aspirin, 600mg clopidogrel and was started on intravenous unfractionated heparin and emergently transported to the cardiac catheterization laboratory for STEMI. Coronary angiography revealed 100% occlusion of the mid-left anterior descending artery (mLAD) (Figure 3A). Intervention allowed for successful balloon angioplasty with canalization of the mLAD occlusion. However, there was distal embolization of thrombus in the distal, apical left anterior descending artery and persistent no-flow phenomenon despite aggressive balloon angioplasty and intracoronary vasodilators (Figure 3B). Due to persistent haziness around the original mLAD occlusion, there was a decision to perform drug-eluting stent placement. 

In the coronary care unit (CCU), twenty-four hours post catheterization, the patient developed new-onset aphasia. MRI brain without contrast revealed acute infarct of the left frontal cortex and subcortical white matter consistent with ischemic stroke. The patient remained hemodynamically stable and was medically managed. Transthoracic echocardiogram (TTE) revealed an ejection fraction between 40-45% with severe hypokinesis of the anteroseptal, apical-inferior, and septal walls. The patient was medically optimized with "double therapy" (clopidogrel 75 mg daily, apixaban 2.5mg every 12 hours), beta-blocker (metoprolol succinate 100mg daily), angiotensin-converting enzyme inhibitor (enalapril 5mg daily), and a high-intensity statin (atorvastatin 80md daily).

The patient was safely discharged from the CCU to subacute rehab. Subsequently, two weeks later at his telemedicine cardiology follow-up visit, the patient reported he was symptom-free and feeling well. There were no changes to his medical management. 

Case 2

A 91-year-old African American female presented to the ED for intermittent right-sided chest pain radiating to the back. Past medical history was significant for uncontrolled hypertension, which was diagnosed in her early 40's. Per chart review, there was no other pertinent history for cardiac disease. The chest pain started an hour before arrival at the ED and was partially relieved by 81mg aspirin. 

**Figure 4:**
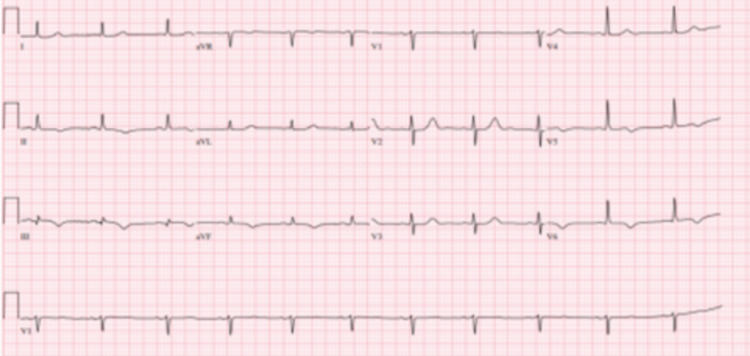
EKG: T-wave inversions in the inferior leads II, III, aVF, and precordial leads V5-V6.

Initial vitals demonstrated a blood pressure of 163/67 mmHg, heart rate 69 beats/minute, respiratory rate 24 cycles/minute, SpO_2_ 97% on room air, temperature 99.6 °F. The patient was well-appearing, alert, oriented, and in no acute distress. The remainder of the physical exam revealed a regular heart rhythm, and no murmurs were appreciated. Lungs were clear to auscultation, and no lower extremity edema. Initial labs demonstrated an elevated brain natriuretic peptide (BNP) and rising troponins (Table 2) . The chest radiograph showed no acute process. An EKG showed normal sinus rhythm with new T-wave inversions in the inferior leads II, III, aVF, and precordial leads V5-V6 (Figure 4). TTE revealed an ejection fraction between 60% with inferior and inferoseptal hypokinesis. 

**Table 2:** Laboratory Values; Case Presentation 2

Case #2			
Hospital Day #	Day 1	Day 2	
Lab Value	-	-	Reference Range
White Blood Cell (WBC)	8.97	8.1	3.50 - 10.80 K/uL
Hemoglobin (Hg)	12.2	12.6	14 - 18 g/dL
Platelet Count (Plt)	212	188	130 - 400 K/uL
Serum Sodium (Na^+^)	136	133	136 - 145 mmol/L
Serum Potassium (K^+^)	4.7	4.6	3.5 - 5.1 mmol/L
Serum Chloride (Cl^-^)	103	101	98 - 107 mmol/L
Serum Magnesium (Mg^+2^)	2.1	-	1.9 - 2.7 mg/dL
Serum Calcium (Ca^+2^)	7.9	8.1	8.2 - 10 mg/dL
Serum Bicarbonate (HCO3^-^)	17	19	21 - 31 mmol/L
Blood Urea Nitrogen (BUN)	41	36	7 - 25 mg/dL
Serum Creatinine (Cr)	1.9	1.4	0.7 - 1.3 mg/dL
Troponin-I (TNI)	2.53	16.07	< 0.15 ng/dL
Brain Natriuretic Peptide (BNP)	298	-	< 100 pg/mL
Serum Lactic Acid	2.1	-	0.5 - 2.2 mmol/L
Venous Blood Gas pH (VBG)	7.32	-	7.31 - 7.41
Serum D-Dimer	105	-	< 499 ng/mL
Thyroid-Stimulating Hormone (TSH)	3.12	-	0.38 - 4.70 uIU/mL

In the ED, the patient received 325mg of aspirin, 300mg clopidogrel, and was started on intravenous unfractionated heparin. The patient was urgently taken to the cardiac catheterization lab for a coronary angiogram and possible intervention for an admitting diagnosis of NSTEMI.

**Figure 5:**
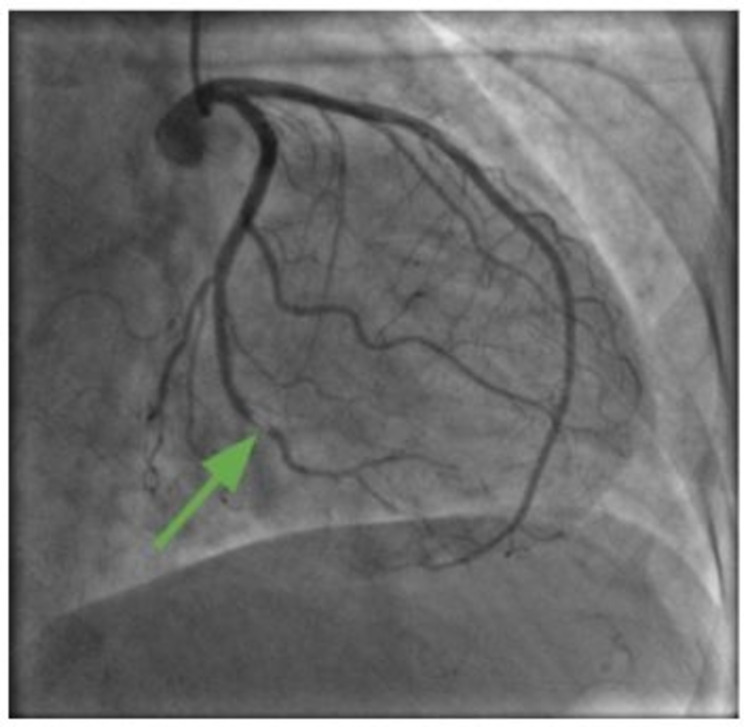
Coronary angiography revealed occlusion of the distal second obtuse marginal (OM2) artery (green arrow).

Coronary angiography revealed occlusion of the distal second obtuse marginal (OM2) artery (Figure 5). The smooth morphology of the lesion and otherwise normal coronaries revealed a possible embolic phenomenon rather than in-situ plaque rupture and thrombosis due to the distal location of the lesion and tortuous coronary anatomy. The interventional cardiology team recommended medical management. 

In the CCU, the patient continued to receive dual antiplatelet therapy (aspirin 81mg daily, clopidogrel 75mg daily) and was started on a high-intensity statin (rosuvastatin 40mg daily), angiotensin-converting enzyme inhibitor (lisinopril 5mg daily), and a beta-blocker (metoprolol tartrate 50mg twice daily). On further questioning, she said that she had been having episodes of palpitations over the past several months that relieved spontaneously. The etiology of her coronary artery embolism and palpitations may have been paroxysmal atrial fibrillation. She underwent loop recorder placement to monitor for an underlying arrhythmia. 

The patient was safely discharged from the coronary care service to her home. Unfortunately, the patient was lost to follow-up.

## Discussion

Atrial fibrillation increases the risk of thrombus formation in the left atrial appendage via Virchow’s triad, which includes three predisposing risk factors which are hypercoagulability, stasis, and endothelial injury. Thromboembolic events often occur during episodes of fibrillation or within the first ten days following cardioversion back to sinus rhythm [1]. Most emboli will be directed towards the central nervous system, with coronary thromboembolism seen as a rare occurrence due to the hemodynamic characteristics of the circulatory system [2]. 

Traditionally, atrial fibrillation has been linked to ischemic stroke but is also associated with type-1 myocardial infarction (T1MI) and type-2 myocardial infarction (T2MI) [3]. Additionally, atrial fibrillation is associated with inflammation that may promote a prothrombotic state and subsequent acute myocardial infarction [1]. 

Coronary artery embolism (CAE) secondary to atrial fibrillation is an under-recognized etiology of ACS. It is a significant cause of AMI with non-obstructive coronary arteries (MINOCA). Before 1960, infective endocarditis accounted for more than half of all cases; however, the rate has declined since then [3]. Contemporary data published by Shibata demonstrated that the most frequent cause of CAE was atrial fibrillation explained in part by the increasing prevalence of AF in an aging population [4]. 

The exact prevalence of CAE is unknown. An extensive review by Shibata et al. of the National Cerebral and Cardiovascular Center (NCVC) database analyzed 1,776 new-onset acute myocardial infarctions between 2001 and 2013 and identified CE (2.9%) in 52 patients with diagnosis through angiography, histology, and imaging [5]. 

Atrial fibrillation remains the most common cause of coronary thromboembolism, however, other potential mechanisms exist, including but not limited to left ventricular mural thrombus from a prior myocardial infarction, aortic and mitral prosthetic heart valves, and dilated cardiomyopathy [1,4,5]. 

The differential diagnosis for MINOCA is broad and includes epicardial spasm, eccentric plaque, Takotsubo syndrome, microvascular spasm, myocarditis, and coronary artery embolism to mention a few [6]. To date, there are no guidelines to diagnose coronary artery embolism, which is why Shibata et al. proposed criteria to standardize the diagnosis [5]. Conventional workup may include cardiac magnetic resonance imaging (CMRI), ventilation-perfusion (VQ) scan, intravenous ultrasound (IVUS), and/or optical coherence tomography (OCT), and a transesophageal echocardiogram (TEE) with agitated saline [1,6].

In 2015 the American College of Cardiology (ACC) published focused guidelines stating that the use of bailout aspiration thrombectomy in patients undergoing primary percutaneous coronary intervention (PCI) is not well established and should only be performed in select cases [7]. In case 1, successful reperfusion was achieved by balloon angioplasty of the mid-left anterior descending artery (mLAD). We considered aspiration thrombectomy, however, due to the distal nature of thrombi embolization, we didn’t attempt it in our case. The TASTE and TOTAL trials compared routine manual thrombus aspiration followed by PCI and PCI alone. Neither trial showed benefit from thrombus aspiration on mortality, rehospitalization for myocardial infarction, and stent thrombosis [7]. 

To date, the 2020 ACC expert consensus pathway for anticoagulant and antiplatelet therapy in patients with atrial fibrillation atrial fibrillation or venous thromboembolism (VTE) undergoing percutaneous coronary intervention (PCI) or with atherosclerotic cardiovascular disease, state that the use of “triple therapy” (dual antiplatelet therapy plus anticoagulation) is not recommended for most patients due to an increased risk of bleeding. If triple therapy is needed, a short duration (e.g., no more than 30 days) is recommended. When combined with an anticoagulant, clopidogrel is the recommended antiplatelet agent for most patients [8]. 

Finally, as there are no clear cardiology guidelines for CAE arrhythmia workup, we recommend continuous cardiac rhythm monitoring for all patients. As demonstrated in case 2, the patient underwent successful placement of an implantable loop recorder (ILR). 

## Conclusions

In conclusion, ACS due to CAE in patients with atrial fibrillation is an underreported finding amongst interventional cardiologists. Knowing that atrial fibrillation is the number one cause of CAE, we must continue to address the root cause of the disease to prevent the onset and mitigate its catastrophic complications.
